# Thoracic Endovascular Aortic Repair for a Ruptured Mycotic Aortic Pseudoaneurysm Secondary to Esophageal Carcinoma

**DOI:** 10.1155/2022/5489653

**Published:** 2022-01-06

**Authors:** Sean-Tee J. M. Lim, Stephen Murphy, Said Atyani, Michael Anthony Moloney

**Affiliations:** ^1^Department of Radiology, University Hospital Limerick, St Nessan's Rd, Dooradoyle, Co. Limerick V94 F858, Ireland; ^2^Department of Vascular Surgery, University Hospital Limerick, St Nessan's Rd, Dooradoyle, Co. Limerick V94 F858, Ireland

## Abstract

A 47-year-old female presented to the emergency department with new episodes of hematemesis. She had a background of unresectable T4b + N1 + M0 esophageal squamous cell carcinoma. Contrast CT thoracic aorta diagnosed a ruptured mycotic aortic pseudoaneurysm of the descending aorta, forming a life threating aorto-esophageal fistula secondary to neoplasm. Due to the high risk of fatal haemorrhage, she underwent successful emergency thoracic endovascular aortic repair (TEVAR). Mycotic aortic pseudoaneurysms are a rare and often fatal complication of esophageal carcinomas. They represent a small subsection of aorto-esophageal fistulas. Early diagnosis with cross sectional imaging and vascular control of the sentinel bleed is essential for survival. TEVAR may be used as a bridge to palliative treatment in the case of unresectable esophageal carcinoma.

## 1. Introduction

Aorto-esophageal fistulas (AEF) are a rare cause of hematemesis, representing around 10% of all aorto-enteric fistulas [1]. They are often fatal when they rupture, leading to massive haemorrhage and sepsis. AEF can occur primarily when due to local disease in the aorta or esophagus, including aneurysms, malignancies, and ulceration. AEF may also occur secondary to damage caused by the presence of foreign bodies, prosthetic grafts, and stents [2]. Here, we report the successful management of AEF caused by a ruptured mycotic aortic pseudoaneurysm secondary to esophageal carcinoma.

## 2. Case Report

A 47-year-old female presented to the emergency department with new episodes of hematemesis. She was recently diagnosed four weeks prior with midesophageal squamous cell carcinoma, stage T4b + N1 + M0. The tumour was deemed unresectable at multidisciplinary meeting due to tumour location being attached to carina and main stem bronchus.

She described three to four episodes of hematemesis occurring that day, with fresh blood and clots. She was haemodynamically stable on admission with heart rate of 100 bpm and blood pressure of 110/80 mmHg. Laboratory results revealed a haemoglobin level of 7.7 g/dL, dropping from 10.9 g/dL three days prior, INR of 1.2, platelets of 274 × 109/L, and C reactive protein of 36 mg/dL. She had a jejunostomy feeding tube due to the esophageal tumour causing dysphagia. The patient was fluid resuscitated and given two units of concentrated red cells bringing her haemoglobin level to 9.1 g/dL.

**Figure 1:**
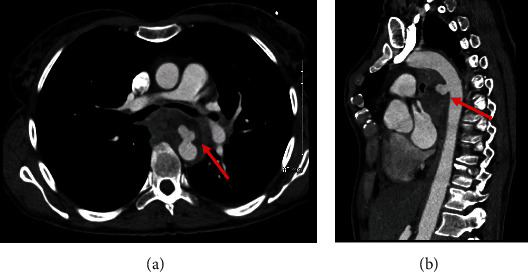
Axial (a) and sagittal (b) CT angiogram images demonstrating extravasation of contrast anteriorly into the surrounding esophageal tumour.

**Figure 2:**
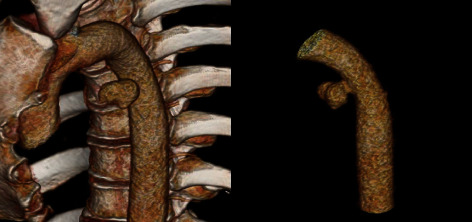
3D reconstruction of the CT images demonstrates the pseudoaneurysm in the descending aorta with its relation to the osseous structures, as well as in isolation.

Contrast CT thoracic aorta revealed a mycotic pseudoaneurysm in the proximal descending thoracic aorta (Figure 1). This was causing an aorto-oesophageal fistula at the level of T6, where the locally invasive esophageal lesion was located. CT 3D reconstruction images demonstrate the shape and location of the pseudoaneurysm (Figure 2).

**Figure 3:**
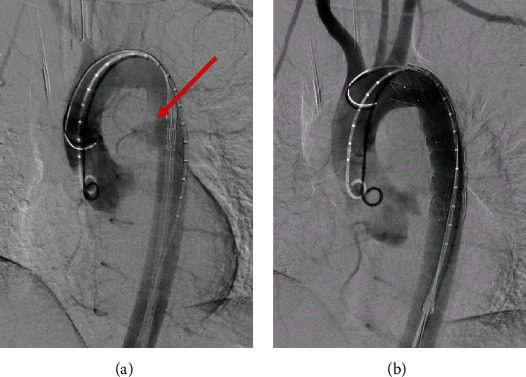
Intraoperative digital subtraction angiography during TEVAR. (a) Prestent deployment showing real time extravasation of contrast (red arrow). (b) Postthoracic stent deployment with successful endovascular sealing.

Due to high risk of rupture with fatal bleeding and the patient's wishes for operative intervention, she was brought to operating theatre that night. CT whole aorta was performed for thoracic endovascular aortic repair (TEVAR) operative planning. Surgery was performed under general anaesthetic with unfractionated heparin was given intraoperatively. The thoracic stent utilized was 96 mm length, 25 mm proximal graft diameter, and 25 mm distal graft diameter (Valiant Navion 2525C96TE). The graft was oversized approximating 10% proximally and was deployed distal to the left subclavian artery. The aim was to seal in normal aorta proximal and distal to the 9 mm defect. With this in mind, we had approximately 43 mm proximal and 43 mm distal to defect which was visible intraoperatively. We did not balloon the stent as we felt this would be contra-indicated given the potential for dissection. Final intraoperative digital subtraction angiography showed an excellent stent position and seal with no filling of the pseudoaneurysm (Figure 3).

**Figure 4:**
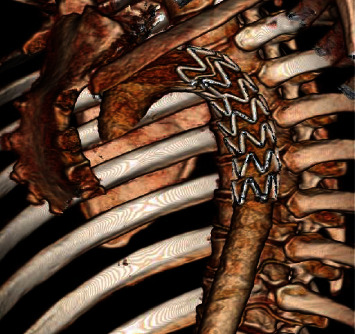
Postoperative CT image reconstruction demonstrating graft location and the successful occlusion of the pseudoaneurysm.

She was admitted to HDU postoperatively with no acute complications, no further hematemesis, and was hemodynamically stable (haemoglobin = 10.7 g/dL). Follow-up CT with image reconstruction demonstrated the successful occlusion of the pseudoaneurysm with a patent graft (Figure 4). She was treated with intravenous antibiotics for 6 weeks followed by a regular course of oral antibiotics as advised by microbiology, due to risk of stent infection. Her care was later taken over by the oncology service, and she was treated with palliative radiation therapy allowing her to live a further twelve months.

## 3. Discussion

Mycotic pseudoaneurysms are irreversible vascular dilatations which occur secondary to infection in the arterial wall. Given the term pseudoaneurysm as the lead to a locally contained hematoma between the tunica media and the tunica adventitia. The term “mycotic” is derived from the mushroom-like gross appearance of the aneurysms and not microbiological reasons [3]. Staphylococcus and Streptococcus species are the most common causative pathogens, with infection occurring secondary to a primary carcinoma [4]. The invasion of esophageal carcinoma into the thoracic aorta is a rare etiology of AEF (17%) with untreated thoracic aneurysm (54.2%) and foreign body ingestion (19.2%) more commonly occurring [5].

The diagnosis of mycotic aortic aneurysms can be greatly challenging due to the common nonspecific symptoms including the presence of fever and general malaise. AEF may present with the classical clinical symptoms known as Chiari triad which is characterised by central chest pain, dysphagia, and sentinel haemorrhage followed by exsanguinating hematemesis [6]. The imaging modality of choice for detection of AEF is multislice CT angiography, with 3D reconstructions being demonstrated in this case. CT findings display a contrast enhancing saccular-shaped aneurysm which may be associated with a periaortic soft tissue mass, oedema, or abscess [7].

There are few published case reports with successful management [8, 9]. Upper endoscopy should be performed in the setting of active hematemesis, whereas CT is the initial imaging study of choice for assessing AEF in for hemodynamically stable patient. After the diagnosis is confirmed, patients should be treated immediately due to the risk of haemorrhage and uncontrollable infection. Management involves initial control the thoracic aorta with open surgery or now more commonly endovascular repair. Open repair usually involves replacement or bypass of the thoracic aorta with resection and repair of the esophagus. The operative mortality of open AEF repair ranges from 45.4% to 55% [8] and thus was not used in this case. TEVAR provides essential haemostasis, acting as a bridge to definitive esophagectomy, or as in this case palliative radiation therapy [10]. It has been reported to have a high technical success rate (87.3%) and favourable 30-day mortality rate (19.7%) when compared to open techniques [11]. In this case, endovascular repair was well tolerated and prevented life threatening hematemesis. On review of previous cases as well as local microbiology advice, long-term antibiotic treatment was used to prevent stent graft infection and sepsis [9].

## 4. Conclusion

Clinicians should have a high index of suspicion for aorto-esophageal fistula for patients presenting with hematemesis on a background history of esophageal malignancy. We recommend a low threshold for CT angiography for patients presenting this way, with early involvement of the vascular and upper gastrointestinal surgical teams. TEVAR for AEF in the setting of unresectable esophageal cancer provides an excellent option for minimally invasive control of haemorrhage, along with a reduced mortality rate compared to open surgical repair.

## Data Availability

All data is available in the text.
